# Gastrointestinal Perforation with Blunt Abdominal Trauma in Children

**DOI:** 10.3390/children11060612

**Published:** 2024-05-21

**Authors:** Victoriya Staab, Srividya Naganathan, Margaret McGuire, Jamie M. Pinto, Harpreet Pall

**Affiliations:** 1 Department of Surgery and Pediatrics, K. Hovnanian Children’s Hospital at Jersey Shore University Medical Center, Neptune, NJ 07753, USA; victoriya.staab@hmhn.org (V.S.); srividya.naganathan@hmhn.org (S.N.); margaret.mcguire@hmhn.org (M.M.); jamie.pinto@hmhn.org (J.M.P.); 2 Department of Surgery and Pediatrics, Hackensack Meridian School of Medicine, Nutley, NJ 07110, USA

**Keywords:** abdominal trauma, children, gastrointestinal tract injury, bowel perforation

## Abstract

Gastrointestinal tract perforation is uncommon in children, accounting for <10% of cases of blunt abdominal trauma. Diagnosis of bowel perforation in children can be challenging due to poor diagnostic imaging accuracy. Intra-abdominal free air is found only in half of the children with bowel perforation. Ultrasound findings are nonspecific and suspicious for perforation in only two-thirds of cases. A computer tomography (CT) scan has a sensitivity and specificity of 50% and 95%, respectively. Surgical decisions should be made based on clinical examination despite normal CT results. Management of bowel perforation in children includes primary repair in 50–70% and resection with anastomosis in 20–40% of cases.

## 1. Introduction

Traumatic injuries are the leading cause of morbidity and mortality in children in the United States [1]. Unintentional injuries account for 6.1%, 32% and 40.8% of mortalities in age groups <1 year, 1–9 years and 10–24 years, respectively [2]. Abdominal trauma is the third most common cause of mortality after brain and thoracic injuries but is often not immediately recognized due to the co-occurrence of other injuries [3]. Common mechanisms leading to blunt abdominal trauma include motor vehicle accidents (MVA), bicycle and sports injuries, non-accidental trauma and fall from heights [3]. In contrast to adults, blunt abdominal trauma accounts for more than 90% of abdominal injuries in children with only a small percentage due to penetrating trauma [4]. Non-accidental trauma represents a quarter of cases of blunt abdominal injury in infants and requires a high index of suspicion to enable timely diagnosis [5]. Solid organ injuries of the liver and spleen account for more than 50% of cases in pediatric blunt abdominal trauma with a smaller percentage secondary to kidney and pancreatic injuries [6,7]. After the liver and spleen, bowel and mesenteric injuries are the third most frequent sites accounting for 10–12% of all cases of blunt abdominal trauma [8,9]. Clinical examination findings pertaining to abdominal trauma may be masked due to depression of consciousness secondary to co-existing traumatic brain injury [3]. This may lead to a delay in the diagnosis of a significant bowel injury. Furthermore, findings of peritonitis can be delayed in children due to the presence of other concomitant injuries [7,8,9,10]. 

Diagnosis of bowel perforation in children can be challenging as imaging modalities typically obtained have poor diagnostic accuracy [8,9,10,11,12]. Intra-abdominal free air is found only in half of the children with bowel perforation [8]. Ultrasound findings such as bowel wall thickening, intramural hematoma and peritoneal fluid are nonspecific and suggestive of perforation in 60–70% of cases [8]. A CT scan is considered the gold standard in cases of abdominal trauma and has a low sensitivity of 50% with a specificity ranging from 78 to 95% [8,9,10,11]. Non-specific CT findings such as intraperitoneal fluid, bowel wall thickening, mesenteric fat streaking and mesenteric hematoma do not reliably predict bowel perforation [13]. Ionizing radiation from CT scans also increases the lifetime risk of developing both solid organ cancers as well as leukemia; therefore, the risks and benefits should be carefully considered prior to obtaining the study [14]. Decisions for laparoscopy or laparotomy should be made based on clinical findings and serial abdominal examination even with normal CT scan results. Management of bowel perforation in children consists of primary repair or resection with anastomosis [11]. 

The aim of this review article is to discuss epidemiology, clinical presentation, diagnostic imaging modalities with their inherent challenges and surgical management options for gastrointestinal tract injury and perforation in children sustaining blunt abdominal trauma. 

## 2. Materials and Methods

A literature review was performed by the two primary authors using Medline, PubMed, Cochrane reviews and Google Scholar databases. Key words used in the search included ‘abdominal trauma’, ‘children’, ‘gastrointestinal tract injury’ and ‘bowel perforation’. Recent as well as historical literature were reviewed by both primary authors for topic applicability, methodology and clinical relevance prior to inclusion. Non-English publications were excluded, as were those that did not include pediatric patients or blunt abdominal trauma mechanisms (i.e., penetrating trauma). Research articles and reviews were prioritized, followed by case series. Only a single case report was included, due to novel presentation. 

The age of patients selected in the studies had wide variability with most of the reviewed articles including age groups between 1 and 17 years with mean ages of 9–10 years. The articles pertaining to non-accidental trauma included infants (<1 year). Some of the studies included adult patients which were primarily used for comparative purposes. 

## 3. Results and Discussion

### 3.1. Epidemiology

Unintentional injury is the predominant cause of death among children aged 1–19 years in the United States, with MVAs being the most frequent etiology [15]. While widespread use of safety restraints for motor vehicle passengers has contributed to a 41% decrease in death rates over time [15], the so-called “seatbelt sign” where bruising is present over the chest and abdomen in the distribution of a safety belt has been associated with blunt abdominal trauma and injury to abdominal organs, inclusive of hollow viscera [16,17,18]. Pediatric blunt trauma accounts for 80–90% of pediatric abdominal injuries [7] and is most frequently associated with MVAs, but other mechanisms have been well described, such as injury secondary to bicycles, the so-called “handlebar injury”, falls onto blunt objects, sports and non-accidental trauma [3,19,20,21]. Importantly, abdominal injury has been described as “the most common cause of unrecognized fatal injury in children” [22]. 

Non-accidental abdominal trauma is likely underreported, is estimated to occur in 0.5–11% of physical abuse victims and can lead to fatal injuries such as bowel perforation in 13–53% of cases [23,24]. In children under 9 years old, abdominal trauma accounts for 3.8–4.3% of child abuse hospitalizations and 4.8–5.9% of abdominal trauma admissions [23]. As opposed to patients with non-inflicted abdominal trauma, patients who are victims of abusive abdominal trauma are more likely to be younger (2 vs. 5.4 years), from racial and ethnic minority groups, have public insurance and live in zip codes with low income [23]. Victims of abusive abdominal trauma seldom have abdominal bruising present on physical exam, so providers need to have a high index of suspicion, or injuries can easily be missed [24]. 

Bowel perforation is a known but uncommon complication of blunt abdominal trauma, with intraperitoneal small bowel injury being most common, accounting for approximately 70% of intestinal injuries [8,9]. The proximal jejunum and the distal ileum are common locations of injury due to proximity to fixed structures (ligament of Treitz and the ileo-cecal valve). Colonic injuries account for about 20% of injuries and duodenal injuries account for about 10% [8]. The exact incidence of bowel perforation in pediatric trauma has not been well described. A large prospective study which included over 12,000 children who suffered blunt trauma to the torso found that <1% suffered injury to the gastrointestinal tract [25]. Another study performed by the Pediatric Surgery Research Collaborative which specifically reviewed blunt intra-abdominal injury found that only 2.7% of 2188 included children who presented with abdominal pain had a bowel injury identified [16]. 

Different mechanisms have been described that may contribute to hollow viscus injury after blunt abdominal trauma: (1) crushing of the bowel between vertebral bone and anterior abdominal wall musculature by blunt force (2) shearing of mobile and fixed bowel segments during brisk deceleration and (3) bursting of viscera from a rapid increase in intraluminal pressure [8,26]. In the event of a deceleration injury, the mesentery of the bowel can be injured and devascularized leading to delayed presentation of bowel perforation [8]. Duodenal injuries are associated with pancreatic injuries due to the compression of both structures against the spine from a lap belt [8,18]. 

### 3.2. Clinical Presentation 

Intra-abdominal injury requiring acute intervention can have variable and unreliable clinical presentation [3]. Symptoms and signs on presentation include vomiting, abdominal pain and tenderness, distention, with decreased or absent bowel sounds and in some cases seatbelt sign, abdominal wall abrasion and ecchymosis [21,25,27,28,29,30,31]. Multisystem trauma with distracting injuries and/or low Glasgow Coma Scale (GCS) can inhibit the provider’s ability to assess for other injuries, including intra-abdominal injury [3,8]. Elevated heart rate is not uncommon and can be from blood loss, pain or stress [20]. Physical exam findings of peritoneal irritation such as rebound tenderness are only elicited in half of patients with bowel injury in the absence of distracting injury and often have late-onset presentation [8,26]. Unfortunately, even a normal abdominal examination in the absence of a distracting injury cannot entirely exclude bowel injury and can lead to a delay in diagnosis, highlighting the importance of serial physical examination when caring for patients with blunt abdominal trauma [10,20,29,32,33].

### 3.3. Diagnostic Evaluation

Diagnostic imaging is critical in pediatric blunt abdominal trauma and has high utility as it relates to solid organ and musculoskeletal injury; however, it has significant limitations in assessing and diagnosing intestinal trauma.

Diagnostic imaging modalities available to assess for an abdominal injury in a child with trauma include focused assessment with sonography for trauma (FAST) scan, plain radiographs, ultrasound, CT, and magnetic resonance imaging (MRI). Invasive diagnostic evaluation options include diagnostic peritoneal lavage (DPL), diagnostic laparoscopy, and exploratory laparotomy [11].

#### 3.3.1. Plain Radiography

Abdominal and upright radiography can occasionally diagnose intestinal injury by the presence of extraluminal air, which only occurs in up to 33% of cases. A change in bowel caliber can also be suggestive of bowel injury [11,34].

#### 3.3.2. FAST

Ultrasonography has limited utilization as a screening tool for intestinal injury. This is due to the low sensitivity of 33% despite a high specificity of 95%. A positive FAST examination often reveals free fluid, which does not differentiate between etiology from solid or visceral organ injury. In a hemodynamically stable patient, a positive FAST can be managed nonoperatively with serial clinical examinations. However, a positive FAST with hemodynamic instability will often require operative exploration to identify the source of free fluid. Hence the utility of this study is limited for accurate diagnosis of hollow viscus injuries. Studies show increased utility of FAST in adult hospitals (96%) as compared to (15%) in children’s hospitals [7,35,36].

#### 3.3.3. CT

The gold standard for diagnosing solid organ injury in trauma is CT, but its role in diagnosing hollow viscus injuries is limited. Findings on CT that are more specific for bowel injury include bowel wall interruption, extravasation of enteric contents and parietal hematoma. These findings have an almost 100% specificity if visualized but have poor sensitivity ranging from 7 to 15% [8]. Non-specific findings that may suggest a bowel perforation include free intraperitoneal or retroperitoneal air, bowel wall thickening or enhancement, mesentery infiltration and free fluid (>25 mL in pediatric patients) without evidence of solid organ injury [8]. A study looking at the correlation of intraperitoneal free air and bowel perforation had a sensitivity of 50% and specificity of 95% [12]. In another study of 2114 pediatric patients with blunt abdominal trauma and CT findings, the presence of more than one nonspecific finding increased the positive predictive value but with decreasing sensitivity [13]. With respect to intraperitoneal fluid, a study of 670 children with blunt trauma showed that the quantity of free fluid (as determined by the number of locations) on initial CT predicted the need for operative intervention [16]. In a review performed of the Cochrane Library database, MEDLINE, and EMASE that evaluated 2596 pediatric patients with significant blunt abdominal trauma, the negative predictive value of abdominal CT was 99.5–99.8% [37]. Despite a negative abdominal CT, five patients clinically worsened progressing to requiring surgical exploration for a missed bowel injury [12,37].

#### 3.3.4. MRI

Research and utilization of MRI is limited in the setting of acute trauma. Access to readily available MRI equipment, prolonged duration of the study, cost associated with the imaging and interpretation, and the need for sedation in younger patients are all limitations of this imaging modality in the setting of a suspected bowel injury. Extraluminal air suggestive of bowel perforation is also difficult to identify on MRI. The usefulness of MRI is more significant in identifying concomitant injuries such as pancreatic and bile duct injuries that can raise suspicion for a bowel injury. In addition, duodenal intramural hematomas may be better identified on MRI compared to CT [38].

#### 3.3.5. DPL

DPL is an invasive diagnostic tool that has fallen out of favor in diagnosing intra-abdominal trauma. It carries a sensitivity of 96–99% and is 98% specific in diagnosing an intra-abdominal injury. DPL has a complication rate of 1% and requires sedation in small children [39]. In a 1996 study, 10 pediatric patients underwent a DPL, half of which were diagnostic of an injury [32]. With the increased sensitivity and specificity of CT scans and the utilization of the FAST examination in the setting of blunt abdominal trauma, the utility of DPL is questionable and has largely been abandoned.

#### 3.3.6. Standardized Grading System

A standardized grading system for describing visceral traumatic injuries has been described by Moore et al. (Table 1, Table 2, Table 3 and Table 4) (reprinted from Moore et al. with permission) and implemented as standard by the American Association for the Surgery of Trauma (AAST) [40]. This standardization helps with communication with respect to the severity of injury and helps guide management.

The gold standard for determining if a trauma patient has sustained a bowel injury in the setting of non-diagnostic imaging but a high clinical suspicion is serial abdominal examinations; the experienced physician should be vigilant as not to delay intervention if the clinical examination is worsening. Delayed recognition of bowel injury could lead to devastating consequences of infection and sepsis and carries a high risk of mortality [29,32].

### 3.4. Management

Management of traumatic intestinal injuries should always start by following the Advanced Trauma Life Support protocols. The initial evaluation should focus on ‘airway, breathing, circulation’ by confirming patent airway, bilateral breath sounds and obtaining accurate blood pressure measurements. Abdominal tenderness and abdominal wall bruising is important to document on the initial exam and may provide clues to bowel perforation. It is important to have good intravenous (IV) access with preferably two large bore IVs to ensure rapid administration of crystalloids and colloids. Patients with hemodynamic instability should receive an initial bolus of 20 mL/kg of crystalloid fluid and repeated if improvement in vital signs are not documented. If after a second bolus the patient remains hypotensive, packed red blood cells 10 mL/kg should be considered. Patients with continued hemodynamic instability, free intraperitoneal air, penetrating abdominal injuries or evisceration of intraperitoneal contents need emergent exploration. Patients who do not require emergent exploration should then undergo diagnostic imaging. In the event of negative imaging, patients with high concern for intestinal injury should be admitted and monitored with serial abdominal exams. Kerrey et al. found 16 children out of 3819 had an intra-abdominal injury despite negative imaging and six of those needed an intervention [41]. Singh et al. found 13 cases of isolated mesenteric injuries. Seven of the 13 developed peritonitis after a period of observation prompting exploration [42].

#### 3.4.1. Operative Exploration

Abdominal exploration is indicated to identify an intra-abdominal injury in the setting of positive or suspicious diagnostic imaging for hollow viscus organ injury or high clinical suspicion either with hemodynamic instability or concerning abdominal examination findings.

#### 3.4.2. Laparoscopy versus Laparotomy

Laparotomy and exploration has been the standard of care for emergent traumatic intra-abdominal injuries for decades. With the advancement of laparoscopic skills, there has been an increasing interest in using laparoscopy in acute trauma. Laparoscopy requires hemodynamic stability as the process involves the creation of a pneumoperitoneum which can affect ventilation and hemodynamic status. However, it is a safe and feasible option for diagnosis and potential therapeutic intervention in the setting of bowel injury in children. In a study conducted between 2004 and 2008, out of 113 children that required abdominal exploration, 28% had a laparoscopy and 19% had laparoscopic interventions [43,44]. Swedniman et al. conducted a review of the National Trauma Data Bank to evaluate the use of laparoscopy in pediatric abdominal trauma. They identified 4554 patients who had operative exploration and 355 (7.8%) had laparoscopy [45]. Of those who had laparoscopy, 18.6% were converted to laparotomy [45]. Patients who underwent laparoscopy were older, had a lower Injury Severity Score (ISS), higher GCS and were more likely to have sustained penetrating trauma. The use of laparoscopy was more common at university hospitals and ACS level I pediatric trauma centers [43]. The use of laparoscopy did increase over the course of their study period from 6.2% in 2010 to 10.1% in 2014. Negative laparotomy has a complication rate up to 41% [46]. Laparoscopy can be considered in a selected population to aid in the diagnosis of intestinal injury and possible repair.

Exploratory laparotomy is the standard of care in patients with hemodynamic instability or respiratory compromise in the setting of pediatric abdominal trauma with a concern for intra-abdominal injury. Exposure allows full exploration of solid and hollow intra-abdominal organs as well as access to gain vascular control for hemorrhage. Damage control temporizing maneuvers can also be employed, such as temporary abdominal packing and leaving an open abdomen with intestinal discontinuity as needed. This allows for an opportunity to adequately resuscitate and prevent the lethal triad of hypothermia, coagulopathy, and acidosis [47].

#### 3.4.3. Stomach

Operative exploration should be complete to not miss any small injury. A systemic stepwise approach is needed. The stomach should be mobilized to evaluate the posterior wall by opening the lesser sac. Grade I or II lesions can be debrided to healthy edges and then repaired primarily in one or two layers. Grade III injuries can be sutured or closed with the use of staplers. It is important to note the location of the pylorus and to avoid narrowing or occluding its lumen. A pyloroplasty should be considered if the wound involves or is adjacent to the pylorus [48].

#### 3.4.4. Duodenum

Trauma to the duodenum can be a challenge both in terms of identifying the injured segment and the repair. The first segment is the only portion that is intraperitoneal. Delay in diagnosis directly correlates with mortality and morbidity. Lucas et al. found that mortality increased from 11% of those identified in the first 24 h vs. 40% for those identified after 24 h [49]. Low-grade duodenal injuries are mostly hematomas and superficial lacerations without perforations. They are seen in children with blunt trauma. They can often be managed nonoperatively, but if there is a high suspicion, further evaluation should be performed. Low-grade duodenal injuries can present as progressive gastric outlet obstruction typically 48–72 h after injury. Only 25% of patients with gastric outlet obstruction will present with a duodenal hematoma on imaging [50]. High-grade duodenal injuries are usually seen on CT imaging. Operative exploration should be performed emergently in these cases. There is debate about the repair of high-grade duodenal injuries. In 2019, Ferrada et al. published a series of 372 patients with duodenal injuries and 80% (299) were primarily repaired [51]. Primary repair should again involve debridement of any damaged mucosa and repair in two layers. Care should be taken not to narrow the lumen of the duodenum. The patient should be kept nil per mouth with gastric decompression. Weale et al. report primary duodenal repair in 91 patients where seven had a postoperative leak [52]. Those with a leak were then treated by gastrojejunostomy and pyloric exclusion or secondary repair and drainage.

#### 3.4.5. Small Bowel

The small bowel is the most common site of intestinal injury. The small bowel should be evaluated by starting at the ligament of Treitz. The bowel should then be carefully inspected on both sides in small sections until the cecum. The mesentery should be inspected for any hematomas and injuries along the mesenteric border. Partial thickness small bowel injuries should be closed in a seromuscular layer. Full thickness injuries can be managed by primary closure in one or two layers (interrupted or running), after debridement of necrotic or non-viable tissues. It is key to have a tension-free repair by suturing in a transverse manner to prevent narrowing or stenosis. If there are multiple injuries in a short segment it is better to treat them as a higher-grade injury and perform resection rather than repair. Grades III, IV and V injuries are treated with resection. Repair with hand sewn or stapled anastomosis has been shown to have similar complications [48]. In the event of a hemodynamically unstable patient, necrotic tissue should be resected, and repair delayed to a second look operation.

#### 3.4.6. Colon

Colon injuries can have serious consequences given the degree of bacterial contamination. Primary repair or diversion with colostomy are both options. Treatment is dictated by the severity of the injury: Grade I-III-non-destructive; grade IV and V destructive. Primary repair can be considered for these injuries performed in two layers in a transverse fashion. The use of staplers may also be considered depending on the surgeon’s preference. Repair of grade IV and V injuries involves resection and anastomosis or resection and colostomy. Sharpe et al. reported an algorithm where nondestructive injuries are primarily repaired. Patients with destructive injuries, with hemodynamic instability, comorbidities or who have received greater than 6 units of blood underwent diversion. Patients without the abovementioned findings underwent resection and anastomosis. The use of this algorithm was associated with a decreased rate of abscess formation and trauma-related mortality [53].

## 4. Conclusions

Abdominal trauma in children is primarily due to blunt injuries, with MVAs accounting for a majority of the cases. Bowel perforation is rare in children and accounts for <10% of all cases of abdominal trauma. Diagnostic imaging modalities including CT do not have high sensitivity and may lead to delayed diagnosis. Late recognition of bowel injury could lead to devastating consequences of infection and sepsis and carries a high mortality. Bowel injuries in the setting of blunt trauma can evolve over the course of several days, especially with devascularization injuries. Therefore, clinicians should maintain a high degree of clinical suspicion for hollow viscus injury in a child with trauma and suspicion of bowel injury in the setting of non-diagnostic imaging. Serial abdominal exams should be performed by clinicians well-versed in the recognition of early stages of peritonitis and surgical exploration as needed. In the setting of clinical uncertainty, diagnostic laparoscopy should be considered for definitive diagnosis in the stable pediatric patient with suspected bowel injury.

## Figures and Tables

**Table 1:** Duodenum injury grading.

Grade	Injury Description
I	Hematoma involving single portion of duodenum, laceration partial thickness, no perforation
II	Hematoma involving more than one portion, laceration < 50% of circumference
III	Laceration involving 50–75% of circumference of D2, disruption 50–100% of D1, D3, D4
IV	Laceration > 75% of circumference of D2, involving ampulla or distal common bile duct
V	Laceration massive disruption of duodenopancreatic complex, devascularization of duodenum

**Table 2:** Small bowel injury grading.

Grade	Injury Description
I	Contusion or hematoma without devascularization, partial thickness, no perforation.
II	Laceration < 50% of circumference.
III	Laceration > 50% of circumference without transection
IV	Transection of small bowel
V	Transection of the small bowel with segmental tissue loss, devascularized segment.

**Table 3:** Colon injury grading.

Grade	Injury Description
I	Contusion or hematoma without devascularization, partial thickness, no perforation.
II	Laceration < 50% of circumference.
III	Laceration > 50% of circumference without transection
IV	Transection of colon
V	Transection of colon with segmental tissue loss, devascularized segment.

**Table 4:** Rectum injury grading.

Grade	Injury Description
I	Contusion or hematoma without devascularization, partial thickness laceration
II	Laceration < 50% circumference.
III	Laceration > 50% circumference.
IV	Full thickness laceration with extension into the peritoneum.
V	Devascularized segment
